# Interventions for caregivers of persons with young-onset dementia in hospital environments: A systematic scoping review

**DOI:** 10.1177/13872877261469822

**Published:** 2026-08-05

**Authors:** Joan R. Fang, Adam P. Vogel, Dennis Velakoulis, Yiqing Yuan, David Nayler, Samantha M. Loi, Lynette Joubert

**Affiliations:** 1 Department of Social Work, 2281 The University of Melbourne, Melbourne, Australia; 2 Neuropsychiatry Centre, Royal Melbourne Hospital, Melbourne, Australia; 3 Department of Audiology and Speech Pathology, 2281 The University of Melbourne, Melbourne, Australia; 4 Department of Psychiatry, 2281 The University of Melbourne, Melbourne, Australia; 5 Department of Social Work, Fudan University, Shanghai, China

**Keywords:** Alzheimer's disease, caregiving, frontotemporal dementia, psychosocial interventions, young-onset dementia

## Abstract

**Background:**

Caregivers of patients with young-onset dementia (YOD), most commonly Alzheimer's disease and frontotemporal dementia, face unique challenges balancing employment, childcare, finances, and complex care responsibilities. Tailored interventions delivered in hospital environments may provide timely support and optimize caregiver preparedness, but evidence regarding these interventions remains limited.

**Objective:**

To map and synthesize evidence on interventions supporting caregivers of individuals with YOD within hospital environments offering diagnostic services, multidisciplinary care, and opportunities for caregiver engagement. Intervention types, delivery modes, outcomes, and evidence gaps will be highlighted.

**Methods:**

A systematic scoping review adhering to PRISMA-ScR guidelines was conducted. Medline, Embase, PsycINFO, EBM Reviews, SocINDEX, CINAHL, and Scopus were searched for studies published up to 2025. Peer-reviewed studies in English describing or evaluating interventions for YOD caregivers were included. Data on study characteristics, intervention type, delivery, outcomes, and key findings were extracted and synthesized narratively.

**Results:**

Eight studies met the inclusion criteria. Interventions were largely focused on psychoeducation and support groups. Most studies targeted spouses with few including other caregiver types. Interventions addressed caregiver functions such as task management, safety, information, and caregiver needs related to emotional support and burden reduction, but rarely were both comprehensively addressed. Quantitative outcomes were mixed while qualitative findings consistently reported improved preparedness, support, and coping.

**Conclusions:**

Evidence on caregiver interventions in hospital environments is limited and largely exploratory. Future work should embed supports within diagnostic pathways, incorporate structured assessment, tailor interventions by subtype and caregiver role, and use outcome measures sensitive to early, meaningful changes.

## Introduction

Young-onset dementia (YOD) is defined as dementia with symptom onset before age 65, and represents approximately 9% of all dementia cases worldwide.^1^ Compared to older-onset dementia (OOD), YOD encompasses a broader etiological spectrum, including Alzheimer's disease (AD), frontotemporal dementia (FTD), vascular dementia, and less commonly familial forms, metabolic or autoimmune conditions, and other secondary causes.^2^ Genetic contributions are more common compared to OOD, particularly in FTD, with approximately 15% of YOD FTD cases having a family history.^2^ The global prevalence of YOD has more than doubled over the past three decades, from approximately 1 million in 1990 to 2.28 million in 2021, most probably reflecting improved recognition and diagnostics rather than demographic shifts.^3^

People with YOD often present during a life-stage characterized by active employment, family responsibilities, and financial commitments, creating a unique set of psychosocial stressors for families.^4–6^ Spouses often have to assume full-time caregiving roles, whilst children take on responsibilities prematurely.^4,5^ Older parents as well as siblings may also provide significant support.^4,5^ These role shifts, combined with hereditary concerns about risk and future planning can destabilize family structures and exacerbate emotional and financial strain.^2,4,5^ Behavioral and psychological symptoms, which are especially marked in FTD, further intensify caregiving challenges and contribute to high levels of distress, anxiety, depressive symptoms, and social isolation, compared to caregivers of persons with OOD.^7–9^ The limited availability of peer support and age-appropriate services for younger families exacerbates these challenges.^4,10,11^

Caregiver interventions, including counselling, psychoeducation, peer support, skills training, and respite care, are well established in OOD, but these programs often assume that the caregiver is retired and has fewer competing responsibilities.^7,12,13^ Caregivers of individuals with YOD are frequently subsumed into older adult focused services despite clear evidence that their needs, coping strategies, and service utilization patterns differ substantially.^4,14^ The Joint Solutions Project, the largest YOD study in Australia, highlighted fragmented service provision, wide variability in diagnosis and follow-up care, and limited ongoing support.^15,16^ Specialist and age-appropriate services were associated with improved continuity, care quality, and caregiver satisfaction.^15,16^ The findings of specific gaps in care were echoed by international studies.^11,14,17,18^

While interventions for caregivers of persons with YOD have been evaluated primarily in community settings, hospital environments, including inpatient care, outpatient clinics, and memory clinics remain critical yet unexplored contexts.^19,20^ These settings are important, representing early points in the care trajectory, where families face diagnostic uncertainty, recurrent crises, and transition between services.^2,13,21^ Hospital encounters can amplify caregiver stress, as families must navigate complex decisions, coordinate multidisciplinary care, and advocate for appropriate treatment or discharge with limited guidance.^17,18^ Early-stage interventions in hospital settings, even modest ones, have been shown to improve caregiver well-being, reduce anxiety, and enhance caregiving capacity.^13,21^

Integrating caregiver support interventions within hospital environments offers distinct advantages. Embedded, multidisciplinary interventions can streamline care coordination, reduce service fragmentation, and improve engagement and continuity.^2,13,21,22^ Multidisciplinary teams including neurology, psychiatry, social work, and allied health can ensure that interventions are developmentally appropriate, targeted, and respond to the unique needs of YOD families.^2,13,21,22^ Such models align with evidence that proactive and integrated support can mitigate caregiver distress and optimize outcomes for both caregivers and patients.^2,13,21,22^ Despite the potential for hospital interventions, structured programs tailored to the specific needs of YOD families remain uncommon.^19^

This systematic scoping review aims to examine caregiver interventions in the hospital environment to systematically map existing strategies, identify gaps in support, and synthesize lessons that can inform the integration of developmentally appropriate caregiver-focused supports into hospital-related services. This approach complements the existing evidence and knowledge base by highlighting how hospital and health services can contribute to continuity of care and address unmet needs for patients and families.

## Review question

A systematic scoping review with the primary aim of describing the evidence available to better understand the impact of caregiver interventions in hospital environments on caregivers of people with YOD was undertaken. The secondary aim of the review was to identify gaps in knowledge to address areas for future research and practice development. The review was guided by the Arksey and O’Malley^23^ Scoping Review Framework, providing a structured approach to address the stated aims.

The review question guiding this study was: What is the impact of caregiver interventions in hospital environments on caregivers of people with YOD? For this review, hospital environments are defined as inpatient wards, outpatient clinics, memory clinics, and other hospital-affiliated services where caregiver interventions are delivered. The objectives were to:

- map out and describe the types and characteristics of caregiver interventions in hospital environments available in the literature.
- summarize the reported outcomes of these interventions on caregivers of people with YOD.
- identify gaps in the evidence base to guide future research, clinical practice, and service development.

By focusing on hospital environments, this review seeks to clarify how and where caregiver support is currently integrated into clinical care for YOD, and to highlight opportunities for more responsive, targeted interventions within healthcare systems.

## Inclusion criteria

All peer-reviewed studies, regardless of study design, published in English, and available up to the final search date (15 December 2025) were eligible for inclusion.

### Participants

Articles focusing on caregivers were included. Caregivers were defined as informal caregivers, including unpaid family members and friends of persons diagnosed with YOD. Articles focused on caregivers of persons with OOD were excluded, unless data for YOD caregivers were separately reported. Studies which focused only on patients with YOD, without any involvement of family and caregivers, were excluded.

### Concept

Articles evaluating, discussing, and describing interventions, programs, or services aimed at supporting caregivers were eligible. Interventions included, but were not limited to education, psychosocial support, training, counselling, respite, or navigation services. Articles were excluded if they only described caregiver experiences without reference to an intervention or support mechanism.

### Context

Interventions delivered in hospital environments, including but not limited to acute care wards, memory clinics, neurology clinics, psychiatry departments, or other specialized units, were eligible for inclusion. Hospital environments were defined as sites providing diagnostic assessment and specialist care for YOD, where individuals and their families present and continue to receive services. Interventions delivered exclusively in community settings such as home-based, aged care facilities, or community support groups, without any hospital involvement, were excluded. Studies where the setting was unclear or mixed, and hospital-specific data could not be extracted, were excluded.

### Types of sources

Peer-reviewed studies including quantitative, qualitative, mixed-methods, program evaluations, quality improvement studies, as well as descriptive reports of caregiver support models, were eligible. However, editorials, commentaries, letters to the editor, or opinion pieces without empirical data were excluded.

## Methods

A systematic scoping review methodology was used to map the evidence on caregiver interventions in hospital environments for families affected by YOD. This approach was selected due to the small and heterogeneous body of literature which included varied intervention types, clinical settings, and outcome measures. A traditional systematic review of effectiveness was not feasible and risked overstating conclusions, whereas a scoping review allowed clarification of concepts, identification of gaps as well as research priorities. This approach maintains methodological transparency through a structured search strategy, clear eligibility criteria, and systematic data charting, while accommodating the exploratory aims of the review.

The review utilized the five-stage framework outlined by Arksey and O’Malley,^23^ which includes: 1) identifying the research question, 2) identifying relevant studies, 3) study selection, 4) charting the data, and 5) summarizing and reporting the results. The review was also conducted in accordance with the JBI Manual for Evidence Synthesis and the Preferred Reporting Items for Systematic Reviews and Meta-Analyses Extension for Scoping Reviews (PRISMA-ScR) guidelines,^24,25^ ensuring rigor, transparency and methodological consistency. These frameworks provide a structured approach to map heterogeneous research findings and are widely recognized for their contribution to quality and consistency of scoping reviews.

The protocol for this review was prospectively registered on PROSPERO CRD420251111287, enhancing transparency and reducing the risk of bias.

### Search strategy

A comprehensive search was conducted across Medline, Embase, PsycINFO, EBM Reviews, SocINDEX, CINAHL, and Scopus to capture the multidisciplinary nature of YOD caregiver intervention research, which spans neurology, psychiatry, social care, and allied health. The Medline strategy (Table 1), developed in consultation with an academic librarian and senior researchers with expertise in YOD and caregiver interventions to ensure accuracy, completeness, and optimal database coverage, was adapted for all other databases. No date limits were applied, but only English language, peer-reviewed studies were included to ensure methodological rigor, given the variability and uneven quality of grey literature in this area. Search terms were intentionally broad due to the heterogeneous terminology used to describe YOD and caregiver interventions, maximizing sensitivity and reducing the risk of missing relevant studies. Reference lists of included articles were also hand-searched.

**Table 1.** Search strategy for individual databases medline (ovid) searched on 27 June 2025.

Ovid MEDLINE(R) ALL <1946 to June 27, 2025>			
#	Concept	Search String	N
1	Young-onset dementia	(Early onset dementia or EOD or Young onset dementia or Younger onset dementia or YOD or Frontotemporal dementia or FTD or Huntington disease or Young onset alzheimer or Early onset alzheimer).mp. [mp = title, book title, abstract, original title, name of substance word, subject heading word, floating sub-heading word, keyword heading word, organism supplementary concept word, protocol supplementary concept word, rare disease supplementary concept word, unique identifier, synonyms, population supplementary concept word, anatomy supplementary concept word]	30238
2	Caregiver intervention	((Famil* or Carer* or Caregiver* or Partner* or Spouse* or Guardian* or Children*) adj4 (Intervention* or Program* or Therap* or Group* or Training* or Activit* or Service* or Strateg* or Project* or Support* or Treatment*)).mp. [mp = title, book title, abstract, original title, name of substance word, subject heading word, floating sub-heading word, keyword heading word, organism supplementary concept word, protocol supplementary concept word, rare disease supplementary concept word, unique identifier, synonyms, population supplementary concept word, anatomy supplementary concept word]	392666
3	Hospital environments	(Hospital* or Health service* or Memory Clinic* or Department* or Medical Cent* or Inpatient or Outpatient or Tertiary care or Ward*).mp. [mp = title, book title, abstract, original title, name of substance word, subject heading word, floating sub-heading word, keyword heading word, organism supplementary concept word, protocol supplementary concept word, rare disease supplementary concept word, unique identifier, synonyms, population supplementary concept word, anatomy supplementary concept word]	3062688
4		1 and 2 and 3	79

All records were imported into EndNote 21 and subsequently uploaded to Covidence, where duplicate entries were removed. Two independent reviewers (JF and YY) conducted blinded title-and-abstract and full-text screening against pre-specified inclusion and exclusion criteria. Disagreements were resolved through consensus and adjudication by a third reviewer when necessary. Authors were contacted for clarification when required to determine eligibility. Screening decisions, conflict resolution, and reasons for exclusions at full text were documented within Covidence. The review process adhered to PRISMA-ScR guidance to enhance transparency and reproducibility.^24,25^

### Data extraction and synthesis

Data extraction was guided by the review's aim to map caregiver interventions for persons with YOD in hospital environments. A data extraction form was developed and piloted on three studies to ensure relevance of fields, and was refined with input from co-reviewers. Two reviewers (JF and YY) independently extracted data, including author, year, country, study aims, settings, population and sample size, study design, intervention characteristics, key findings, and reported limitations. Disagreements were resolved by discussion and by a third reviewer when required. Extracted data were summarized across two complementary tables. One table detailed key study characteristics and findings (Table 2), and the other outlined intervention characteristics (Table 3), allowing a clear presentation of both the evidence base and intervention features.

**Table 2.** Key study characteristics and findings.

Authors (year), country	Study aims	Study settings	Study population and sample size	Study design and measures	Key findings	Limitations
Bruinsma et al.^32^ (2021), Netherlands JBI Critical Appraisal Checklist for Quasi-Experimental Studies	To evaluate how caregivers perceive tailored content iteratively developed for family caregivers of persons with YOD from an earlier Partner in Balance intervention for family caregivers of persons with dementia who still live at home.	University Medical Centre and Alzheimer Centre	15 spouses, 21 children, 3 siblings, 1 parent. Disease types were 67.5% AD, 22.5% FTD, 7.5% VD, LBD 2.5%. 15 male caregivers and 25 female caregivers. 4 spouses and 11 family members lost to non-completion. Mean age of participants was 50.1 (range 17–74)	Feasibility and usability study utilizing pre-post design. The study utilized a custom Program Participation Questionnaire, role adaptation questionnaire and conducted deductive qualitative content analysis.	The intervention was found to be feasible, usable, and acceptable for caregivers of people with YOD. Participants valued the flexibility, content, and coaching; reported improved self-awareness, preparedness, and coping. Preliminary program effect indicated an increase in role adaptation, especially self-efficacy.	Small sample size and short follow-up. Experience with using web-based technology was not inventoried. Usual care and support provided to informal caregivers and other supports were allowed.
Cañabate et al.^28^ (2025), Spain JBI Critical Appraisal Checklist for Quasi-Experimental Studies	To assess the effectiveness of a support and training group specifically targeted towards spouses of individuals with young onset dementia and its impact on reducing caregiver burden.	Alzheimer Centre	77 of 116 spouses. Disease types were 61% AD, 39% other dementias. 41.6% had mild dementia and 37.7% had moderate dementia. 45.5% male spouses and 54.5% female spouses. Mean age of caregiver participants was 59.7.	Pre-post design. The study utilized the ZBI, CDR, MMSE, and caregiver employment activity. Ethnographic analysis of participants’ narratives, coding into thematic groupings, and triangulation was conducted.	Support groups perceived as beneficial, particularly for those with high initial caregiver burden. Quantitative measures did not show significant reduction in burden. Qualitative findings highlighted improvements in emotional support, understanding of dementia, caregiving strategies, and reduced feelings of loneliness.	Findings may have limited generalizability as it was conducted within the cultural context of Catalonia. Small sample size and methodological constraints restricted depth of analysis and generalizability.
Climans et al.^34^ (2023), Canada JBI Critical Appraisal Checklist for Quasi-Experimental Studies	To evaluate the impact of piloted support groups on YOD caregivers’ psychosocial wellbeing, coping abilities, and knowledge.	Academic Health Sciences Centre	15 spouses and 16 adult children (AD and FTD); 13 female spouse and 10 female adult children. Mean age of spouse caregiver was 61.1, and mean age of adult children caregiver was 34.4.	Pilot pre-post design, mixed methods study. Standardized tools utilized: PHQ-2, IADCQ, PM, CCS, CAMI, NPI-Q. Demographics and surveys with Likert-scale items and 3 open-ended questions.	Caregivers felt supported and less alone when sharing experiences with other similar caregivers. The intervention demonstrated potential to reduce negative impact of caregiving, particularly among adult children and to improve knowledge and capacity building for caregivers.	Single-site convenience sample; small group sizes and incomplete follow-up limited confidence in findings. Only immediate post-program outcomes assessed; long-term impact of intervention unknown.
Diehl et al.^35^ (2003), Germany JBI Critical Appraisal Checklist for Quasi-Experimental Studies	To 1) provide caregivers with a medical model of FTD, with legal and financial advice, and information on available help, 2) to understand specific issues of FTD caregivers and differences in AD caregiver burden, 3) to encourage mutual support among caregivers and develop coping strategies, 4) to evaluate the intervention.	Hospital Department of Psychiatry and Psychotherapy	8 spouse caregivers, all with FTD and mostly mild dementia. All participants were women. Age range of participants was 46–69.	Single-group pre-post intervention study with a six month follow-up. An adapted questionnaire was administered after the session; a newly developed custom 14-item yes/no survey was administered post follow-up to assess knowledge, coping, burden, self-care, guilt, and support.	Caregivers felt relieved by sharing their problems with others; they could learn from each other and share coping strategies. Caregiver support groups are useful in management of patients with FTD; groups should be tailored to the specific problems and needs of these caregivers – they can be continued in the absence of professional input.	Risk of bias due to small, self-selected sample with no comparison group; descriptive results limit causal inference. Comparison with AD caregiver burden not based on standardized scales.
Holthe et al.^29^ (2018), Norway JBI Critical Appraisal Checklist for Qualitative Research	To explore and understand caregivers’ experiences, challenges, and perceptions regarding the use of assistive technology in young-onset dementia.	Memory Clinics	12 persons with YOD and 13 Family Carers (FC). There were 8 female and 4 male persons with YOD, 4 wives, 6 husbands, 1 mother, 1 daughter, 1 son. Mean age of persons with YOD was 60, with age range 45–64. Mean age of caregivers was 57, with age range 35–78	Single group intervention design with multiple data collection points. Participants were provided AT and interviewed for 1–11 times during the 18-month intervention. The study utilized semi-structured interviews and NVivo-assisted qualitative coding.	The study found benefits for the FC, especially with simply designed AT. Successful use required a committed caregiver throughout the intervention. Users require professional advice and support; occupational therapists may have a significant role in the process. AT interventions must be based on needs of person with YOD and their caregivers, including capabilities, preferences, habits, and coping strategies.	Data collected by six different OTs and quality of OT-participant relationship may have influenced the results. Limited emphasis on collaboration with family carers at the onset of the study may have reduced intervention impact.
Morhardt et al.^30^ (2015), USA JBI Critical Appraisal Checklist for Textual Evidence: Policy	To describe and illustrate the implementation of a coordinated, personalized care model for people with dementia and their caregivers, highlighting the processes, challenges, and practical considerations in delivering integrated care.	Outpatient Clinical Setting	Sample size not provided; participants had YOD, specifically AD, PPA, PCA, bvFTD	Conceptual analysis of a clinical care model (CARE-D) for young-onset dementia. The study provided a conceptual analysis of psychosocial and rehabilitative strategies for care on YOD.	Dementia care should incorporate symptoms and profile rather than be solely diagnosis-based. Tailored interventions that consider individual, family, and social context are key to improving quality of life. Multidisciplinary collaboration is essential for effective implementation.	Interventions rely on availability of trained multidisciplinary specialists, which may limit scalability. Outcome measurement was beyond the scope of this study.
Rosness et al.^31^ (2008), Norway JBI Critical Appraisal Checklist for Analytical Cross-Sectional Studies	To examine the provision of support to patients and family carers of patients with FTD compared with patients and family carers of patients with early onset AD, and their satisfaction with the support they had been given.	Outpatient Memory Clinics and Neurology Departments	60 dyads (23 FTD, 37 AD patients and carer; 61% spouse 26% children, 13% others). 17 FTD and 16 AD patients were women. Mean age of patients was 59.5.	Comparative cross-sectional study conducted ∼2 years after diagnosis. Statistical analysis using demographic information, care provision, caregiver, and satisfaction variables.	Caregivers of patients with FTD were significantly less satisfied with the provision of information about the disease compared with carers of early-onset AD patients. They were also significantly less satisfied with counselling, and follow-up advice.	Small sample size and convenience sampling limit generalizability. Carer satisfaction measured via interviews at variable times post-diagnosis.
Shnall et al.^33^ (2013), Canada JBI Critical Appraisal Checklist for Textual Evidence: Policy	To explore how the needs of families living with FTD resulted in three program initiatives developed to meet the needs of this population.	Academic Health Sciences Centre	Virtual support group: 12 spousal caregivers of FTD patients (6 per group). Day program evaluation: 4 FTD patients. Website development: 14 children (aged 11–18) of FTD patients in Skype focus groups.	Descriptive service development study with preliminary feasibility and qualitative evaluation. The study had three embedded services, utilizing a custom observational tool for FTD patients, semi-structured interviews for caregiver and child focus groups.	Services developed with stakeholders in FTD from the onset to address gaps in services. Participant feedback indicated that support provided was meaningful. Online support groups provide accessibility across geographic barriers. Day programs offer direct patient benefits and caregiver respite.	Small convenience sample for the three services limited generalizability. Novel behavioral tracking tool utilized. Website developed not systematically evaluated.

**Table 3.** Intervention characteristics.

Authors (Year)	Intervention name/Type	Intervention focus/ selection criteria	Intervention provider	Care recipient	Mode/Delivery/ duration	Key features
Bruinsma et al.^32^ (2021)	Partner in Balance (Tailored for YOD) Structured modular program	Psychoeducation, self-management, role adaptation, emotional support, coping skills Caregivers aged ≥15 supporting someone with home-living YOD	Healthcare professionals (dementia case managers, psychologists)	Caregiver (spouse and other family members)	Individual coaching Online 8–10 weeks	Four self-chosen thematic modules, video vignettes, self-reflection, step-by-step change plan, with personal online coaching and feedback. Modules were tailored for spouses and other family members.
Cañabate et al.^28^ (2025)	Support group for spouse Fixed members for group sessions	Emotional coping, stress management, caregiving skills, dementia education Spouses of home living YOD individuals, excluding those in care facilities	Psychologist (specializing in dementia and grouptherapy)	Spousal caregiver	Group facilitation In person Fortnightly sessions	90-min sessions with guided discussions and workshops facilitated by psychologist, with flexible and open-ended topics
Climans et al.^34^ (2023)	Virtual support group for YOD spouse/children Fixed members for group sessions	Emotional support, self-reflection, problem solving, role adaptation, social connection Ontario caregivers of home-living YOD individuals with English proficiency and internet access	Experienced master's-level social worker and registered with the local governing body (Facilitated by two social workers trained in group psychotherapy)	Caregiver (spouse and children)	Group facilitation Online 8–10 weeks	60-min session facilitated by social workers trained in psychotherapy, with client-centered and non-directive approach, focus on sharing experiences, mutual problem solving, and social support.
Diehl et al.^35^ (2003)	Structured FTD support group for spouse Fixed members for group sessions	Education, including medical legal, financial resources, and therapeutic support, including coping and mutual support. Spouses of community-living FTD patients	Physician (psychiatry resident and neurology resident) and social worker	Spousal caregiver	Group facilitation In person 7 weeks	Structured sessions over seven weeks including sharing of personal experiences and a final visit to a nursing home, followed by monthly self-help meeting without professional participation.
Holthe et al.^29^ (2018)	Assistive technology intervention Case management	Functional support, safety, caregiver stress reduction, quality of life Home-living YOD patients and consenting caregivers	Occupational therapists	Caregiver & pwYOD	Dyadic case management In person 3 weeks - 18 months	Assistive devices tailored to individual needs, comprising initial assessment, follow-up, and observation of device use, with support from an occupational therapist.
Morhardt et al.^30^ (2015)	Care Pathway Model for Dementia (CARE-D) Interdisciplinary case management	Functional support, cognitive and language retraining, environmental modification, behavioral management, caregiver education and training YOD patients and families selected for tailored care based on assessments	Multidisciplinary team consisting of behavioral neurologists, neuropsychologists, neuropsychiatrists, and social workers (relying on input from various team members)	Caregiver & pwYOD	Dyadic case management In person Duration as required	Holistic, symptom-specific care pathway, with individualized interventions based on assessments, interdisciplinary team coordination, tailored strategies emphasizing strengths and meaningful activities, supports caregiver implementation and family functioning.
Rosness et al.^31^ (2008)	Support to family carers of patients with FTD Service support	Assessment and referral-based follow-up to access home help, home nursing, short or long-term nursing home, information, guidance, and counselling Mild-moderate AD and FTD patients and caregivers	Specialist psychologist in geriatric psychiatry and a medical doctor	Caregiver & pwYOD	Service support for caregiver and pwYOD. In person Duration as required	Tailored support and guidance, monitoring of care needs and satisfaction, referrals to day care, home care, nursing home, nursing services, and ongoing assessment of caregiver satisfaction.
Shnall et al.^33^ (2013)	Supportive services for families living with FTD Fixed members for group sessions, day program, online support	Emotional support, self-care, stress reduction, behavioral management, social connection, reduce isolation Spouses of FTD patients at Baycrest	Social worker and multidisciplinary team (occupational therapists, nursing staff, social workers, medical staff, day program staff, researchers).	Spousal caregiver & pwYOD	Group facilitation, structured activities, and website Online and in person 10 weeks facilitated sessions, 10 weeks mutual self-help, and day program duration as required	Online group sessions followed by self-help sessions where a facilitator was available on request. Day program provides structured activities such as exercise, art, music to improve behavior. Website provided a platform for family members to receive support.

Given the heterogeneity of study designs, participants, interventions, outcomes, and analytical methods, meta-analysis or direct comparison of intervention effectiveness was not feasible. Simple descriptive statistics, including counts and ranges, were reported to summarize study and intervention characteristics. Thematic analysis was then applied to synthesize patterns across the evidence base, without comparing outcomes. A narrative synthesis was undertaken using an inductive thematic analysis, guided by that of Pearlin et al.^26^ stress process model. NVivo 14 was used to support systematic data management and enhance transparency. Coding was conducted iteratively, with categories and themes developed from the extracted data. The stress process model was used as a framework to aid interpretation, rather than as deductive coding. This involved detailed review of each study, development of coding categories from the data, and identification of recurring themes across the evidence base.

### Critical appraisal

A risk of bias assessment was conducted to evaluate the methodological rigor of included studies and provide context for interpreting the findings. Although formal quality appraisal is not required for scoping reviews, it was undertaken in our study because of the heterogeneous study designs and limited evidence base for caregiver interventions in YOD. This ensures a detailed understanding of the quality of available research.

The Joanna Briggs Institute Critical Appraisal Tools^27^ were applied across study designs, including quasi-experimental studies, cross-sectional studies, qualitative studies, and textual evidence design. To minimize reviewer bias, two reviewers (JF and YY) independently conducted the appraisals, with disagreements resolved through discussion.

Of the eight studies that were included, three were rated as high risk of bias, four as moderate risk, and one as low risk (Table 4). The appraisal did not influence study inclusion or synthesis, but provides transparency and informs interpretation of the evidence, highlighting that most studies were moderate to high risk and therefore, findings should be considered cautiously.

**Table 4.** JBI quality appraisal of included studies.

Checklist for quasi-Experimental studies	Bruinsma et al.^32^ (2021)	Cañabate et al.^28^ (2025)	Climans et al.^34^ (2023)	Diehl et al.^35^ (2003)
Is it clear in the study what is the “cause” and what is the “effect” (i.e., there is no confusion about which variable comes first)?	Yes	Yes	Yes	Yes
Was there a control group?	No	No	No	No
Were participants included in any comparisons similar?	Yes	Yes	Yes	No
Were the participants included in any comparisons receiving similar treatment/care, other than the exposure or intervention of interest?	Unclear	Unclear	Unclear	Unclear
Were there multiple measurements of the outcome, both pre and post the intervention/exposure?	No	Yes	Yes	No
Were the outcomes of participants included in any comparisons measured in the same way?	Yes	Yes	Yes	No
Were outcomes measured in a reliable way?	Yes	Yes	Yes	Unclear
Was follow-up complete and if not, were differences between groups in terms of their follow-up adequately described and analyzed?	No	No	No	Yes
Was appropriate statistical analysis used?	Yes	Yes	Yes	Yes

Checklist for cross-Sectional studies	Rosness et al.^31^ (2008)			
Were the criteria for inclusion in the sample clearly defined?	No			
Were the study subjects and the setting described in detail?	Yes			
Was the exposure measured in a valid and reliable way?	Yes			
Were objective, standard criteria used for measurement of the condition?	Yes			
Were confounding factors identified?	No			
Were strategies to deal with confounding factors stated?	No			
Were the outcomes measured in a valid and reliable way?	Yes			
Was appropriate statistical analysis used?	Yes			

Checklist for qualitative research	Holthe et al.^29^ (2018)			
Is there congruity between the stated philosophical perspective and the research methodology?	Yes			
Is there congruity between the research methodology and the research question or objectives?	Yes			
Is there congruity between the research methodology and the methods used to collect data?	Yes			
Is there congruity between the research methodology and the representation and analysis of data?	Yes			
Is there congruity between the research methodology and the interpretation of results?	Yes			
Is there a statement locating the researcher culturally or theoretically?	Yes			
Is the influence of the researcher on the research, and vice- versa, addressed?	Yes			
Are participants, and their voices, adequately represented?	Yes			
Is the research ethical according to current criteria or, for recent studies, and is there evidence of ethical approval by an appropriate body?	Yes			
Do the conclusions drawn in the research report flow from the analysis, or interpretation, of the data?	Yes			

Checklist for textual evidence	Morhardt et al.^30^ (2015)		Shnall et al.^33^ (2013)	
Are the developers of the policy/ consensus guideline (and any allegiances/affiliations) clearly identified?	Yes		Yes	
Do the developers of the policy/ consensus guideline have standing in the field of expertise?	Yes		Yes	
Are appropriate stakeholders involved in developing the policy/guideline and do the conclusions drawn represent the views of their intended users?	Unclear		Yes	
Are biases due to competing interests acknowledged and responded to?	Yes		No	
Are the processes of gathering and summarizing the evidence described?	Yes		No	
Is any incongruence with the extant literature/evidence logically defended?	Yes		Unclear	
Are the methods used to develop recommendations described?	No		No	

## Results

### Study retrieval

The initial search conducted across seven databases in June 2025 identified 518 studies, with no additional studies identified through hand searching of reference lists. Following the removal of 217 duplicates, 301 were screened at title and abstract level. Of these 301 articles, 61 articles met the eligibility criteria for full-text screening. To ensure the review captured the most recent evidence prior to manuscript submission, the searches were updated on 15 December 2025 using the same databases and search strategies. Records identified in the updated search were deduplicated against the initial results and screened using the same eligibility criteria. A final eight studies were included in the review after checking them against the inclusion criteria.

**Figure 1.**
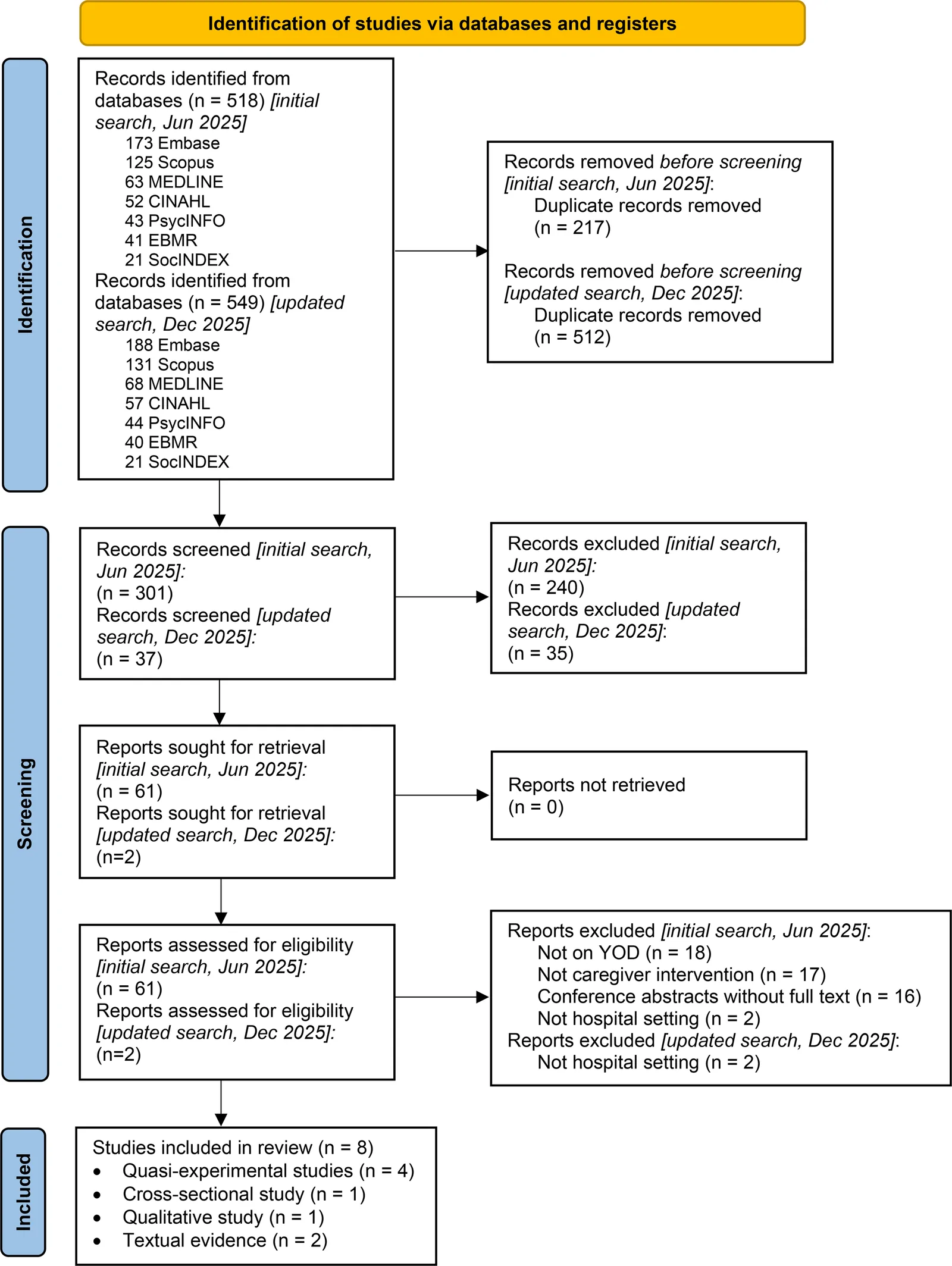
Flowchart identification of studies.

A PRISMA flow diagram is included in Figure 1. Reasons for exclusion were that the articles were not on YOD populations, were not caregiver interventions, or they were not in hospital environments. There were also articles that were conference abstracts without full text; comprehensive searches were conducted using the study title, abstract information, and author details. However, despite these efforts, full-text versions could not be located.

### Characteristics of sources of evidence

Studies were conducted in Canada, Netherlands, Norway, Germany, Spain, and United States. Participants were recruited from or were situated within the following hospital environments: four studies^28–31^ (50%) in specialized dementia centers and memory clinics (Alzheimer center, memory clinic, outpatient clinical setting), three studies^32–34^ (37.5%) in academic or university medical centers (academic health science center, university medical center), and one study^35^ (12.5%) in a hospital department (department of psychiatry and psychotherapy/neurology). Several studies reported more than one setting but were categorized by their primary site of recruitment and intervention. Although studies were conducted across several countries, they provided limited detail on the wider health system, social care, and policy environments in which interventions were delivered. As such, country and setting were extracted as study characteristics, but system-level context was not consistently available for comparison across studies.

### Study focus

Objectives for studies included evaluating interventions, assessing feasibility and perceived usefulness, and understanding caregiver needs and perspectives to inform the design of supportive services. The included studies evaluated a range of caregiver interventions for people with YOD. Some studies assessed structured programs designed to empower caregivers, enhance competence, and promote psychosocial well-being,^32^ while others focused on support or educational groups for spouses, examining their acceptability and perceived value.^28,34,35^ Additional studies explored broader caregiver experiences, including challenges in using assistive technology,^29^ implementation of personalized multidisciplinary care models,^30^ and the specific needs of FTD caregivers, such as differences in burden compared to young-onset AD.^31^ These studies also aimed to provide FTD-specific information to guide coping strategies.^33^

### Study population and sample size

Across the eight studies, 72 persons with YOD and 288 caregivers were represented. Dementia subtype was reported for 219 caregivers across six studies. Within this subgroup, AD was most common (121/219, 55%), followed by FTD (93/219, 43%), with smaller numbers of other dementias (5/219, 2%).^28,29,31–33,35^ Dementia severity was reported in only three studies,^28,31,35^ which described participants as having mild to moderate dementia. Dementia severity was assessed using the Clinical Dementia Rating^28^ and Mini-Mental State Examination^28,31^ in two studies, while the third was reported qualitatively but not formally assessed.^35^ In terms of caregiver type, spouses were the largest subgroup studied (205/288, 75%), followed by children (68/288, 20%), with smaller numbers of siblings (13/288, 4%) and parents (2/288, 1%). Caregiver age, where reported, ranged from 14 to 89 years, reflecting the inclusion of caregivers across multiple kinship roles and life stages, including spouses, children, siblings, and parents who support people with YOD.^28,29,32,34,35^ Across studies reporting caregiver age, the median caregiver age was approximately 57 years, although reported ages varied widely both within and between studies.^28,29,32,34,35^ Inconsistent age reporting across studies precluded reliable calculation of pooled mean ages by caregiver type. Gender was reported for 208 caregivers (115 female, 93 male); two studies did not report caregiver gender,^31,33^ and one intervention-focused study did not include caregiver characteristics.^30^

Sample sizes were generally small, reflecting pilot, feasibility, or support group designs, with individual caregiver studies including 4-116 caregivers^28,32–35^ and dyadic studies 12–60 patient-caregiver pairs,^29,31^ while one intervention description did not include a study sample.^30^ Only one study reported non-completion, with 15 non-completers.^32^

### Study design and methods

Studies included utilized a range of designs and methodological approaches, reflecting the developmental and exploratory state of intervention research for caregivers of persons with YOD. Methodologies comprised several quasi-experimental designs,^28,32,34,35^ a qualitative study,^29^ a cross-sectional study,^31^ as well as two forms of textual evidence as defined by JBI. These were a conceptual description of a multidisciplinary YOD care model,^30^ and a service development study focused on FTD.^33^ Study durations varied substantially. Studies with shorter intervention or follow-up periods typically evaluated structured support groups or modular programs,^32–35^ whereas longer study periods reflected either longitudinal implementation and follow-up of individualized supports^29^ or analyses of longitudinal clinical datasets rather than longitudinal intervention effects.^28,31^

Across the quantitative studies, outcome measures varied widely. Measures assessing the person with YOD included tools capturing dementia severity, including the Mini-Mental State Examination^28,32^ and Clinical Dementia Rating.^28^ Outcomes targeting caregivers also varied widely. Burden, stress, and psychological well-being were assessed using instruments such as the Zarit Burden Interview,^28^ Perceived Stress Scale,^32^ Hospital Anxiety and Depression Scale,^32^ and Patient Health Questionnaire.^34^ Other measures such as Self-Efficacy Scale^32^ and Caregiver Competence Scale^34^ captured caregiver confidence and competence, while the Pearlin Mastery Scale^32^ and Carers’ Assessment Management Index^34^ were used to examine sense of mastery and practical care management. Feasibility and perceived usefulness were assessed using customized instruments including the Program Participation Questionnaire^32^ and an adapted evaluation questionnaire.^35^ This diversity, combined with minimal standardization, limits comparability and may partly explain inconsistent detection of improvements across caregiver outcomes.

Qualitative methodologies were employed to capture caregiver experiences, needs, and contextual factors shaping intervention engagement. Approaches included semi-structured interviews,^29^ content analysis,^32^ and thematic analysis.^28,29,33,34^ These methods played a critical role in exploring the processes underlying intervention outcomes and highlighting psychosocial challenges disproportionately experienced by YOD families, which may not be sufficiently captured by standard quantitative tools. Studies describing multidisciplinary care models and service development initiatives used descriptive and observational techniques to map care processes, examine feasibility, and outline implementation considerations within clinical settings.^30,33^

### Intervention components

Across the included studies, intervention components clustered into two overarching domains that reflect the distinctive care demands of YOD: (a) supporting caregiving functions and (b) addressing caregiver needs. Within each domain, programs integrated varying combinations of educational, functional, behavioral, psychosocial stress management and emotional support elements, although most targeted only a subset of relevant care challenges (see Table 5). Interventions are synthesized collectively to highlight shared caregiver needs across YOD. However, it is notable that clinical heterogeneity, particularly between variants such as AD and FTD, may impose distinct demands on caregivers. Study findings are summarized in Table 2, with intervention characteristics outlined in Table 3.

**Table 5.** Heatmap on intervention focus areas across YOD caregiver studies.

Authors, year	Supporting caregiver functions						Addressing caregiver needs					
	*Caregiving Skills*	*Functional Support*	*Cognitive/language Retraining*	*Behavioral Management*	*Information/Education*	*Psychoeducation*	*Emotional Support*	*Stress Management*	*Self-Care*	*Social Connection*	*Role Adaptation*	*Caregiving Respite*
Bruinsma et al.^32^, 2021						✓	✓	✓	✓		✓	
Cañabate et al.^28^, 2025	✓				✓		✓	✓				
Climans et al.^34^, 2023							✓		✓	✓	✓	
Diehl et al.^35^, 2003				✓	✓	✓	✓	✓		✓		
Holthe et al.^29^, 2018	✓	✓										
Morhardt et al.^30^, 2015	✓	✓	✓	✓	✓	✓						
Rosness et al.^31^, 2008		✓			✓		✓					✓
Shnall et al.^33^, 2013				✓			✓	✓	✓	✓		✓

A related distinction was whether interventions were delivered to caregivers only or to both the person with YOD and the caregiver. Caregiver-only interventions included Bruinsma et al.,^32^ Cañabate et al.,^28^ Climans et al.,^34^ and Diehl et al.^35^ These interventions generally focused on supporting caregivers’ knowledge, coping, emotional adjustment, role adaptation, stress management, and peer connection. They were commonly delivered through online modules, individual coaching, or facilitated support groups. In contrast, dyadic and family-centered interventions by Holthe et al.,^29^ Morhardt et al.,^30^ Rosness et al.,^31^ and Shnall et al.^33^ more often addressed the interactions between the person with YOD, the caregiver, and the service system, including functional support, behavioral management, assistive technology, care coordination, service referral, respite, and multidisciplinary care planning. These distinctions are largely aligned with the two domains identified in this review where caregiver-only interventions more often addressed caregiver needs, while dyadic and family-centered interventions more often supported caregiving functions.

### Supporting caregiving functions

#### Monitoring and patient well-being

Several interventions focused on maintaining day-to-day functioning and safety, which are often heightened in YOD due to rapid disease progression and the behavioral dysregulation seen in syndromes such as FTD.^2,11^ Holthe et al.^29^ evaluated an assistive technology intervention incorporating memory supports, safety devices, and localization tools. Qualitative accounts indicate that caregivers perceived reduced worry and fewer disruptions to daily routines.^29^ However, successful implementation depended heavily on caregiver engagement, familiarity with the devices, and continued professional follow-up, factors particularly relevant in some types of YOD, where loss of insight and disinhibition may undermine consistent use.^29^

Addressing these implementation challenges at a service-delivery level, the CARE-D pathway described an interdisciplinary model informed by comprehensive neuropsychological assessment across cognitive, behavioral, and functional domains, alongside structured psychosocial assessment of patients and caregivers.^30^ These assessments were used to guide tailored caregiver guidance, safety planning, and coordinated follow-up to support continued participation in daily activities across dementia subtypes.^30^ While the CARE-D pathway did not report standardized caregiver outcome measures, it illustrates how structured assessment and coordinated follow-up can be embedded into hospital environments to support monitoring, caregiver involvement, and adaptive care planning in YOD, where heterogeneous presentations require flexible rather than syndrome-specific approaches.^30^

#### Information and training

Information and training-based interventions sought to enhance caregiver knowledge, preparedness, and confidence. Bruinsma et al.^32^ evaluated a modular, web-based self-management program incorporating video vignettes, reflective tasks, goal setting, and personalized coaching. Feasibility, usability, and acceptability were assessed using the Program Participation Questionnaire, with scores exceeding the predefined feasibility threshold across caregiver groups.^32^ Quantitative outcomes were assessed pre- and post-intervention using measures of role adaptation and caregiving self-efficacy.^32^ A statistically significant improvement in care-management self-efficacy was observed among other family caregivers (t(12) = 3.37, p = 0.006), while spouses showed no significant change.^32^ Qualitative Program Participation Questionnaire findings further indicated perceived improvements in preparedness, coping, and confidence, highlighting the value of flexible, accessible formats for working-aged caregivers.^32^

Rosness et al.^31^ compared informational and follow-up support for caregivers of persons with FTD versus those with AD. FTD caregivers reported lower satisfaction with both information received (30% versus 56%, p = 0.05) and counselling provided by specialist services (35% versus 43%, p = 0.05), suggesting that limited clinical expertise in FTD, constrained service resources, and diagnostic delays likely contribute to this gap.^31^ Conventional information-based programs may inadequately address the unique behavioral and emotional complexities of the disease.^31^ These factors highlight the gap in standard information-based programs, which are often insufficient for behavioral and relational challenges that are unique to FTD.^31,32^

### Addressing caregiver needs

#### Burden reduction

Support groups combining educational and therapeutic elements were used to mitigate caregiver burden. Diehl et al.^35^ described a structured group for FTD spouses that provided a dedicated space to discuss behavioral challenges, emotional strain, and specific financial and legal issues. Cañabate et al.^28^ implemented a spousal support group incorporating psychoeducation and stress management strategies. While quantitative outcomes showed no significant changes in burden, qualitative data highlighted enhanced emotional support and reduced isolation.^28^ These short-term benefits are meaningful in YOD, where abrupt role shifts, relational strain, and loss of age-expected life trajectories place younger spousal and family dyads under exceptional pressure.^28,35^

#### Emotional support and social connection

Interventions aimed at emotional support and peer connectedness primarily aimed to reduce isolation and strengthen caregivers’ emotional coping. Climans et al.^34^ conducted a mixed-methods pre-post evaluation of a virtual support group and reported increases in the proportion of caregivers rating coping strategies as helpful on the Carer's Assessment of Managing Index, with post-intervention scores rising from 75% at baseline to 100% across domains for spousal caregivers, whereas among adult children, the increase to 100% was confined to problem-solving strategies, while other coping domains showed smaller changes.^34^ Qualitative findings further indicated improved emotional expression, validation through peer connection, and perceived support, suggesting that even brief virtual support groups may offer meaningful emotional benefits for caregivers of people with YOD.^34^ Consistent with these findings, Shnall et al.^33^ reported qualitative feedback from families involved in a multi-service FTD support model, including online support groups and specialized day programs. Caregivers described these services as relevant for addressing the behavioral and emotional challenges characteristic of FTD, particularly in the context of limited age-appropriate supports.^33^

#### Flexible respite

Only a small number of interventions incorporated respite, despite its recognized importance for YOD caregivers balancing employment, dependent children, and complex behavioral care. Shnall et al.^33^ described a specialized FTD daycare service providing structured activities for persons with FTD while offering caregivers temporary relief. Similarly, Rosness et al.^31^ examined a range of formal and informal respite options, including home help, home nursing, day care programs, supporting persons, and short or longer-term nursing home stays. Caregivers of people with FTD were offered more services than caregivers of young-onset AD, reflecting higher care demands in caring for people with FTD, yet satisfaction with services was noted to be relatively low.^31^ These studies highlight challenges in developing flexible, responsive respite models for YOD, with current options appearing limited and not yet fully aligned with families’ needs. As these studies were conducted more than a decade ago, it remains unclear how respite models have evolved, signaling an important area for updated investigation.^31,32^

## Discussion

### The current state of caregiver interventions in hospital environments

This systematic scoping review synthesized evidence on interventions supporting caregivers of persons with YOD in hospital environments, examining the scope and nature of intervention, caregiver outcomes, and gaps in the evidence base. Across the eight included studies, interventions were delivered in diverse hospital environments, highlighting the potential role hospitals have as integrated settings for caregiver support. This is an important area to examine as caregiver experiences within hospital environments are closely linked to overall patient experience, clinical decision making, care coordination, and transition across services. Supporting caregivers at key hospital touchpoints, particularly around diagnosis and early specialist care, may have benefits that extend beyond caregiver well-being to influence patient engagement, continuity of care, and family confidence in navigating complex health systems. The overall findings reflect the exploratory nature of this literature and the distinct challenges faced by caregivers of people with YOD.

#### Current YOD caregiver intervention models in hospital environments

Caregiver interventions were mostly support groups and psychoeducation programs combining educational and therapeutic components that were valued for providing a structured space to discuss challenges, share experiences, and receive peer support.^28,32–35^ Virtual interventions also demonstrated potential for enhancing emotional expression and connectedness, particularly for caregivers managing employment and family responsibilities.^33,34^ In contrast, respite services, critical for caregivers balancing employment, childcare, and intensive behavioral care, were rarely integrated into interventions within hospital environments. When respite options were described, satisfaction varied, and available services were often perceived as insufficiently aligned with family needs.^31,33^ These findings suggest that current caregiver interventions in hospital environments emphasize psychosocial support and education, with limited attention to functional relief or sustained caregiver capacity. While these models describe what forms of support are currently available, understanding their impact requires closer examination of how outcomes are measured and interpreted.

#### What outcome measures capture and miss in YOD caregiver interventions

The impact of interventions on caregiver outcomes appeared mixed when assessed primarily through quantitative measures. Quantitative findings showed limited or inconsistent improvements, often due to small sample sizes, short intervention duration, and limited sensitivity of standardized tools to detect changes over brief timeframes.^28,31–35^ These measures are typically designed to capture cumulative or longer-term caregiving strain and may therefore be less suitable to brief and early-stage intervention contexts.^12,36^

In contrast, qualitative findings consistently indicated meaningful benefits that caregivers experience soon after the intervention, including enhanced emotional support, reduced isolation, improved preparedness, and increased coping capacity.^28,29,33^ These benefits reflect shifts in understanding of the disease and perceived support rather than an immediate reduction in overall burden or psychological distress.^28,29,33^ This evidence suggests that commonly used outcome measures may be better suited for detecting longer-term caregiver strain, while possibly missing early and meaningful changes that brief or early-stage interventions are most likely to produce, such as a sense of support, guidance, and confidence in navigating services.^12,36^ This distinction is particularly relevant in YOD, where early interventions are often intended to enable caregivers to understand the diagnosis, consider next steps, and navigate services following the diagnosis, rather than immediately reducing overall care burden or psychological distress.^14,17,18^ These outcome patterns raise a broader question about whether YOD caregiver interventions in hospital environments are meaningfully differentiated from those delivered in community settings.

### Community-based interventions versus interventions in hospital environments

While caregiver interventions were delivered through hospital environments, it was unclear whether and how these interventions differed from those offered in community settings. The following sections examine the extent to which hospital environments shaped intervention design, timing, and content.

#### Limited integration into hospital care processes

Although the caregiver interventions were delivered in hospital environments, many closely resembled community caregiver programs, such as psychoeducation and peer support groups.^28,32–35^ Few studies described how hospital-specific processes such as diagnostic disclosure, multidisciplinary assessment, or coordinated follow-up shaped intervention design or delivery.^30^ The similarity to community-based programs and the absence of detail relating to hospital-specific processes indicate that most interventions were delivered as standalone services, rather than embedded into routine hospital care.^28,32–35^

This is important because the uniqueness of a hospital environment is the access to specialist expertise, multidisciplinary teams, and opportunities to align caregiver support with clinical decision making and care planning.^2,17,18^ This may be particularly relevant for caregiver-only and dyadic interventions. Hospitals, memory and neurology clinics, psychiatry departments, and other specialized units often encounter the person with YOD and their caregiver together at key clinical moments, including during inpatient admission, crisis presentations, assessments, diagnosis disclosure, outpatient review, and discharge planning. These encounters provide opportunities to assess what caregivers need and what caregivers are being required to do.

The distinction between caregiver-only and dyadic interventions provides a useful lens for interpreting the two domains identified in this review: addressing caregiver needs and supporting caregiver functions. The caregiver-only format may be better suited for interventions focused on emotional support, peer connection, psychoeducation, and role adjustment, while the dyadic and family-centered format may be more suited to interventions requiring direct attention to functional, behavioral, safety, and service-related needs involving the person with YOD. These latter forms of support may be particularly suited to hospital environments, where clinicians may encounter the person with YOD and caregiver together and have access to multidisciplinary expertise. However, given the small and heterogeneous evidence base, this distinction should be understood as a possible relationship between intervention format and purpose, rather than evidence favoring one model over the other. The limited reporting of hospital-specific processes also constrains conclusions about what hospitals are currently doing differently, and what constitutes best practice in caregiver interventions within hospital environments.

#### The diagnostic and early-post diagnostic phase: a missed opportunity

There is a critical opportunity within hospital environments to provide support for caregivers during diagnosis and early post-diagnostic adjustment, where families often experience shock, uncertainty, and anticipatory grief, alongside needing to make practical and complex decisions.^2,17,18^ Caregivers may be navigating employment, financial implications, parenting responsibilities, safety planning, referral pathways, and in some cases, engagement in clinical trials.^2,17,18^ Despite this, few interventions explicitly targeted the diagnostic and early post-diagnostic phase as a distinct support period. Instead, most interventions focused on ongoing coping, education, and peer support, which are important needs, but can typically be addressed later and can also be supported in community settings.^28,32–35^ This gap highlights a missed opportunity for hospital services to provide timely and phase-specific caregiver support aligned with diagnostic encounters and early adaptation.

#### Limited syndrome-specific caregiver guidance

Most studies did not systematically address differences in caregiver needs across dementia subtypes despite well-established differences between conditions such as AD and FTD.^28–30,32,34^ Behavioral symptoms in FTD in particular, often require more intensive behavioral management guidance and specialized education.^2,10^ These subtype-related differences have direct implications for how caregiver interventions should be designed and delivered.^2,10^ In hospital environments, where specialist diagnostic expertise is concentrated, caregivers may reasonably expect syndrome-specific guidance.^2,10,31^ However, dissatisfaction with information and counselling among caregivers of FTD point to a mismatch between need and service response in some contexts.^2,10,31^ These findings suggest that caregiver interventions in hospital environments have yet to fully tailor support based on the diagnosed dementia subtype.

### Limitations of the existing evidence base

Interpretation of current findings should be considered in light of the methodological limitations of the existing literature. Studies were largely exploratory and quasi-experimental, most commonly single group, pre-post, or pilot feasibility designs, often without control groups and follow-up.^28,29,31–35^ Outcome evaluation was inconsistent; only a subset of studies conducted repeated outcome measurement before and immediately after the intervention,^28,32,34^ while others relied on single post-intervention assessments.^29,31,33,35^ Additionally, only three studies were multisite,^29,31,32^ restricting generalizability beyond single-service settings.

Although these designs are appropriate for early-stage intervention development and service innovation, particularly in a relatively under-research area such as YOD, when combined with small sample sizes, short intervention periods, and heterogeneous outcome measures, they limit the ability to attribute observed changes to the intervention itself, and constrain comparability across studies.^12,36^ Qualitative findings provided valuable insight into caregiver experience but were seldom integrated with quantitative outcomes in ways that clarified how or why change occurred.^29,33^ Differences by caregiver roles, dementia subtype, and socio-cultural context were also rarely examined together, reducing the capacity to assess if interventions were appropriately matched to caregiver needs.^28,29,31,33–35^ Finally, half of the studies were over ten years old. While this may limit the relevance to contemporary hospital care, it also highlights enduring gaps in structured caregiver support within hospital environments.^30,31,33,35^

Additionally, given the international scope of this review, intervention findings should be interpreted with attention to differences in how dementia-related medical care, social care, long-term care, and family support are organized and funded. This is important as countries may organize dementia support differently. Some countries may provide dementia support through government-funded health and social care services, while others may rely more on separate insurance, disability, or longer-term care pathways.^37^ These differences may shape how YOD caregiver interventions are accessed, delivered, and sustained. However, these system-level influences could not be examined in detail, as the individual articles did not consistently describe the wider health system, social care, and policy context surrounding each intervention. Interpretation is also complicated by variation within countries, differences between centers, and changes in dementia care over time. These contextual differences should therefore be understood as considerations for transferability rather than explanatory categories for intervention outcomes.

Beyond the characteristics of the included studies, several methodological considerations of the review should also be acknowledged. This review included only English-language and peer-reviewed studies. While this approach prioritized methodological rigor and transparency, it may have restricted the breadth of evidence captured. Nonetheless, comprehensive multi-database searching, dual independent screening, and structured data extraction and appraisal strengthen confidence in the overall patterns identified.

### Implications for clinical practice, research, and service development

#### Assessment and risk stratification within diagnostic pathways

Structured assessment of caregiver risk and support needs can guide the targeting of interventions, beginning at diagnosis and continuing as caregiving circumstances evolve.^28–30,32^ Across studies, interventions often addressed selected aspects of caregiving, which may reflect how interventions were developed rather than systematically assessed caregiver needs, leaving other relevant support domains unaddressed (Table 5). Structured assessment can identify caregivers with differing risk levels, defined by likelihood of burnout, high emotional stress, caregiving burden, or psychosocial distress, as well as clinical factors such as the stage and trajectory of the person's dementia.^13,22,36,38,39^ Lower-risk caregivers may benefit from brief psychoeducation and navigation support embedded within routine care, whereas higher-risk caregivers, or those supporting individuals at more advanced stages of disease may require prioritized access to more intensive or coordinated support, including more frequent clinical follow-up, targeted psychosocial support, facilitated access to peer support services, or coordination with respite and community-based services.^14,30,38^ Risk-stratified intervention delivery supports efficient allocation of hospital resources, and is a pragmatic approach for specialist services operating under capacity constraints, ensuring that more intensive support is directed to caregivers with greater levels of need.^2,36^

#### Tailored hospital caregiver support across the YOD trajectory

Interventions in hospital environments should account for the diversity of caregiver roles, responsibilities, and capacities.^2,4,11,14,15^ Most studies focused on spouses, who often experience substantial emotional, relational, and practical burdens.^14,17,28,29,33,35^ Other caregiver subtypes, including children, siblings, and parents, remain underrepresented despite facing distinct challenges.^4,14,15^ For example, adult children may juggle employment, childcare, and financial responsibilities alongside caregiving.^4,14,15^ Recognizing these differences is critical to designing interventions that reflect caregivers’ lived experiences and daily realities.^2,4,11,14,15^ Delivery methods should be matched to caregivers’ capacities, preferences, and practical constraints, such as work commitments, childcare responsibilities, and technological literacy.^2,17,30^ The choice between caregiver-only or dyadic formats should also be guided by the purpose of support. Caregiver-only interventions may be more suitable when the focus is on caregiver knowledge, coping, emotional adjustment, or peer connection, while dyadic or family-centered approaches may be needed when support involves communication, behavioral management, practical support, supervision, care coordination, or interactions with the service system.^2,17,30^ Tailoring should also be phase sensitive, with hospitals uniquely positioned to provide structured support during diagnosis and early adjustment, followed by subsequent linkage to longer-term community supports where required and as care demands evolve.^2,17,30^ By tailoring both content and delivery to the specific needs and life contexts of diverse caregiver subtypes, interventions are more likely to be meaningful, acceptable, and effective.^2,17,30^ Embedding structured assessment and tailored support within routine diagnostic and follow-up processes ensures interventions are timely, responsive, and integrated with ongoing clinical care.^2,17,30^

#### Selecting appropriate outcome measures for interventions in hospital environments

The findings in this study also highlight the need to align outcome measures with the goals and timing of caregiver interventions in hospital environments.^12,36^ Early-stage supports delivered around diagnosis are unlikely to produce immediate reductions in burden or psychological distress, but may yield meaningful changes in preparedness, confidence, emotional validation, and navigational capability.^12,36^ Future research should prioritize outcome measures that are sensitive to early, clinically meaningful change, and feasible within hospital environments, alongside longer-term outcomes where sustained impact is expected.^12,36^ Mixed-methods approaches may be particularly valuable in capturing both measurable change and caregiver experience, helping to clarify what works, for whom, and at what point along the caregiving trajectory.^12,36^

## Conclusion

This systematic scoping review suggests that hospitals represent an important point of contact for families affected by YOD, yet caregiver interventions delivered in hospital environments remain limited in number and scope. Existing interventions are most commonly focused on psychosocial and educational support, and in many cases, resemble approaches used in community-based settings. While these supports are valued by caregivers, they are not always clearly integrated within diagnostic and specialist processes or consistently aligned with the specific timing and demands of post-diagnostic adjustment.

Across studies, benefits were more consistently reflected in caregivers’ experiences of emotional support, reduced isolation, improved preparedness, and increased coping capacity, rather than a reduction in burden or psychological distress scores. This pattern highlights the importance of aligning outcome measurement with the goals and expected effects of early-stage interventions in hospital environments, particularly around diagnosis and early care planning. It also suggests that the distinctive potential of the hospital environment lies less in delivering novel intervention formats but more in offering timely, coordinated, and specialist-informed support at critical points of the care pathway.

In summary, the findings of this review highlight opportunities to strengthen caregiver support within hospital environments through clearer integration with diagnostic pathways, structured assessment of caregiver needs, and more tailored support that reflects caregiver roles, disease stage, and dementia subtype. Future research would benefit from studies clearly specifying intervention content and delivery approach in hospital environments, alongside outcome measures sensitive to early and meaningful change. Enhancing caregiver support within hospital environments has the potential to improve continuity of care and better support families and caregivers navigating the challenge of YOD.
